# pH-Dependent Conformational Plasticity of Monoclonal Antibodies at the SiO_2_/Water Interface: Insights from Neutron Reflectivity and Molecular Dynamics

**DOI:** 10.1021/acsami.4c14407

**Published:** 2024-12-12

**Authors:** Zongyi Li, Suman Saurabh, Peter Hollowell, Cavan K. Kalonia, Thomas A. Waigh, Peixun Li, John R. P. Webster, John M. Seddon, Fernando Bresme, Jian Ren Lu

**Affiliations:** † Biological Physics Group, School of Physics and Astronomy, Faculty of Science and Engineering, The University of Manchester, Oxford Road, Manchester M13 9PL, United Kingdom; ‡ Department of Chemistry, Molecular Sciences Research Hub Imperial College, London W12 0BZ, United Kingdom; § Dosage Form Design and Development, BioPharmaceutical Development, BioPharmaceuticals R&D, AstraZeneca, Gaithersburg, Maryland 20878, United States; ∥ STFC ISIS Facility, Rutherford Appleton Laboratory, Didcot OX11 0QX, United Kingdom

**Keywords:** monoclonal antibodies (mAbs), protein adsorption, neutron reflection (NR), molecular dynamics (MD), protein therapeutics

## Abstract

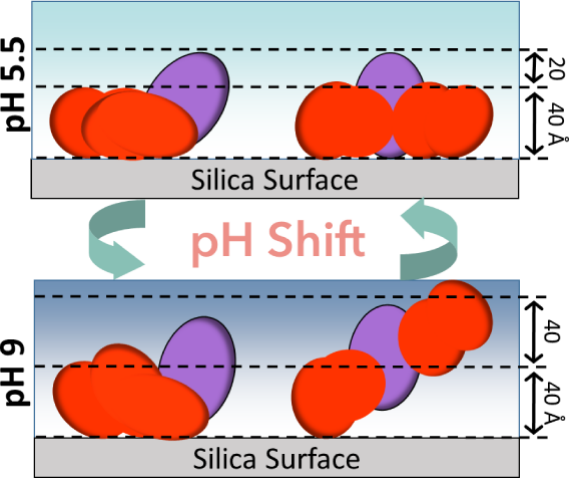

Investigating the molecular conformations of monoclonal antibodies (mAbs) adsorbed at the solid/liquid interface is crucial for understanding mAb solution stability and advancing the development of mAb-based biosensors. This study examines the pH-dependent conformational plasticity of a human IgG1k mAb, COE-3, at the SiO_2_/water interface under varying pH conditions (pH 5.5 and 9). By integrating neutron reflectivity (NR) and molecular dynamics (MD) simulations, we reveal that the mAb irreversibly deposits onto the interface at pH 5.5, with surface density saturation reached at 20 ppm bulk concentration. At pH 5.5, the adsorbed mAb adopts a stable “flat-on” orientation, while at pH 9, it assumes a more flexible conformation and a “tilted” orientation. This pH-dependent orientation shift is reversible and influenced by the distinct surface charge properties of the Fab and Fc fragments, with the Fc fragment more prone to desorption at higher pH. The root-mean-square deviation (RMSD) analysis further shows that COE-3 maintains structural stability upon adsorption across both pH levels, showing minimal unfolding or denaturation. These findings highlight how pH-dependent electrostatic interactions between mAb fragments and the SiO_2_ interface drive conformational adjustments in the intact mAb, offering insights into adsorption-induced aggregation and suggesting pH modulation as a mechanism for controlling biosensor efficiency.

## Introduction

Monoclonal antibodies (mAbs) are a protein class that has revolutionized medicine and biotechnology.^1,2^ Bioengineered mAbs biologics have been employed in interventions for various diseases, including cancer, autoimmune disorders, and infectious diseases.^3^ Additionally, they have played a crucial role in developing advanced biosensors for medical diagnostic and biological analysis.^4^ However, despite their extensive application, the development and use of mAb biologics face significant challenges, particularly protein aggregation and particle formation. While these issues can affect product stability, they also introduce adverse immunogenic responses upon administration if particle levels exceed acceptable limits.^5−9^ A series of studies have identified interfacial protein adsorption as a primary cause of this aggregation and particle formation.^10−12^ Specifically, research has highlighted two pathways of adsorption-induced aggregation: one involves irreversible conformational changes after adsorption at interfaces, which enhances protein–protein attractions and may lead to aggregation in bulk upon desorption.^13,14^ The other pathway suggests that aggregation occurs at the interface, seeded by initially adsorbed proteins.^15,16^ Although the detailed mechanisms behind this process remain largely unexplored, understanding the conformation of the adsorbed protein is critical to unraveling the mechanistic processes in either pathway. Furthermore, insights into the conformation and orientation of the adsorbed mAb at the interface are also essential in developing biosensors. In these mAb-based biosensors, mAbs are immobilized onto substrates through adsorption, where their conformational orientations determine their antigen-binding availability and efficiency.^17^ A favorable orientation of mAb can directly improve the immunosensor performance by 200-fold compared with disoriented immobilization.^18^

This study investigates the conformational plasticity of a human IgG1k mAb, COE-3 (145, 560 Da), at the SiO_2_/water interface under varying pH conditions. The SiO_2_/water interface, akin to a hydrated glass, is a condition mAb biologics encounter throughout their product lifecycle and is also extensively used as the biosensor substrate.^19,20^ Neutron reflectivity (NR) has been a crucial technique for investigating interfacial adsorption of protein due to its ability to provide depth-resolved information on adsorbed layers.^21^ NR can characterize the average surface density of the adsorbed layer as a function of the distance away from the surface, and it is highly sensitive to both the adsorbed amount and the thickness of the adsorbed layer. Some previous studies^12,22−28^ have applied NR on mAb adsorption at the SiO_2_/water interface, inferring the orientations of mAbs by comparing the thickness of the adsorption layer with their crystallographic dimensions, thereby classifying orientations as “end-on”, “flat-on” and others under various adsorption conditions. However, conclusions drawn only from this approach are limited by the structural complexity of the mAbs. Each mAb consists of two antigen-binding fragments (Fabs) and one crystallization fragment (Fc), connected by a flexible hinge region.^29^ The flexibility complicates the interpretation of density functions observed in NR data, particularly when the density profile is multistepped. For example, previous studies^25,26^ have used two or three-stepped density functions to describe the mAb adsorption layer, but it remains unclear whether these varied densities resulted from specific tilting orientations, fragment arrangements, or multilayered adsorption. Furthermore, the lack of isotope-labeled mAb samples adds to the difficulty in distinguishing between the different constituent fragments in the NR studies. Recently, Ruane et al.^30^ demonstrated the use of rigid-body modeling to investigate the orientation of the adsorbed mAb at air/water and oil/water interfaces. However, rigid-body modeling lacks the flexibility to capture dynamic conformational changes in mAbs, limiting its ability to represent realistic adsorption behavior. To address these challenges, this study combines NR and all-atom molecular dynamics (MD) simulations to gain a more comprehensive understanding of the orientation, structural stability, and conformation of adsorbed mAbs. MD simulations offer detailed insights into potential protein conformations under varying pH conditions from a free energy perspective, while NR provides experimental measurements of the structural properties of the adsorbed mAb layer.

The pH is one of the most critical factors impacting the protein interfacial adsorption on a charged surface, such as SiO_2_, which is inherently negatively charged in aqueous solutions. By modulating the electrostatic properties of both the protein and the surface, pH directly affects electrostatic interactions between protein and interface, as well as protein–protein interactions.^31−39^ A previous study^27^ has shown that mAb adsorption peaks at pH values near the mAb’s isoelectric point (pI), primarily due to reduced protein–protein lateral repulsion. However, relying solely on the overall pI and net charge of an mAb can be insufficient for understanding its adsorption behavior and conformational adjustments in response to pH changes. Notably, the Fab and Fc fragments of mAbs have different surface charge characteristics. For instance, in our model IgG1κ mAb, COE-3, the overall pI is 8.44, but the Fab and Fc fragments have pI values of 9.64 and 6.36, respectively. To better understand the roles played by the Fab and Fc fragments in these conformational changes, we investigated and compared the adsorption behaviors of isolated Fab and Fc fragments obtained through the digestion and repurification of COE-3.

In summary, this study addresses the critical need for understanding the pH-dependent conformational plasticity of mAbs adsorbed at the SiO_2_/water interface. By combining NR and MD simulations, we aim to overcome the limitations posed by the mAb structural complexity and provide deeper insights into the role of Fab and Fc fragment charge characteristics in modulating mAb behavior at the interface. Our findings reveal reversible transitions of adsorbed mAbs between “flat-on” and more flexible “tilted” orientations and distinct adsorption behaviors of Fab and Fc fragments in response to pH changes. These findings not only advance our understanding of mAb interfacial behavior but also highlight the potential to fine-tune antibody conformation and orientation through pH modulation, opening a route to develop innovative strategies for mitigating aggregation in mAb biologics and enhancing biosensor performance.^17,23,40^ Furthermore, this pioneering work combining NR and MD simulations sets the stage for novel analytical approaches in investigating protein interfacial behavior.

## Results and Discussion

### Adsorption of COE-3 at the SiO_2_/Water Interface at pH 5.5

**Figure 1:**
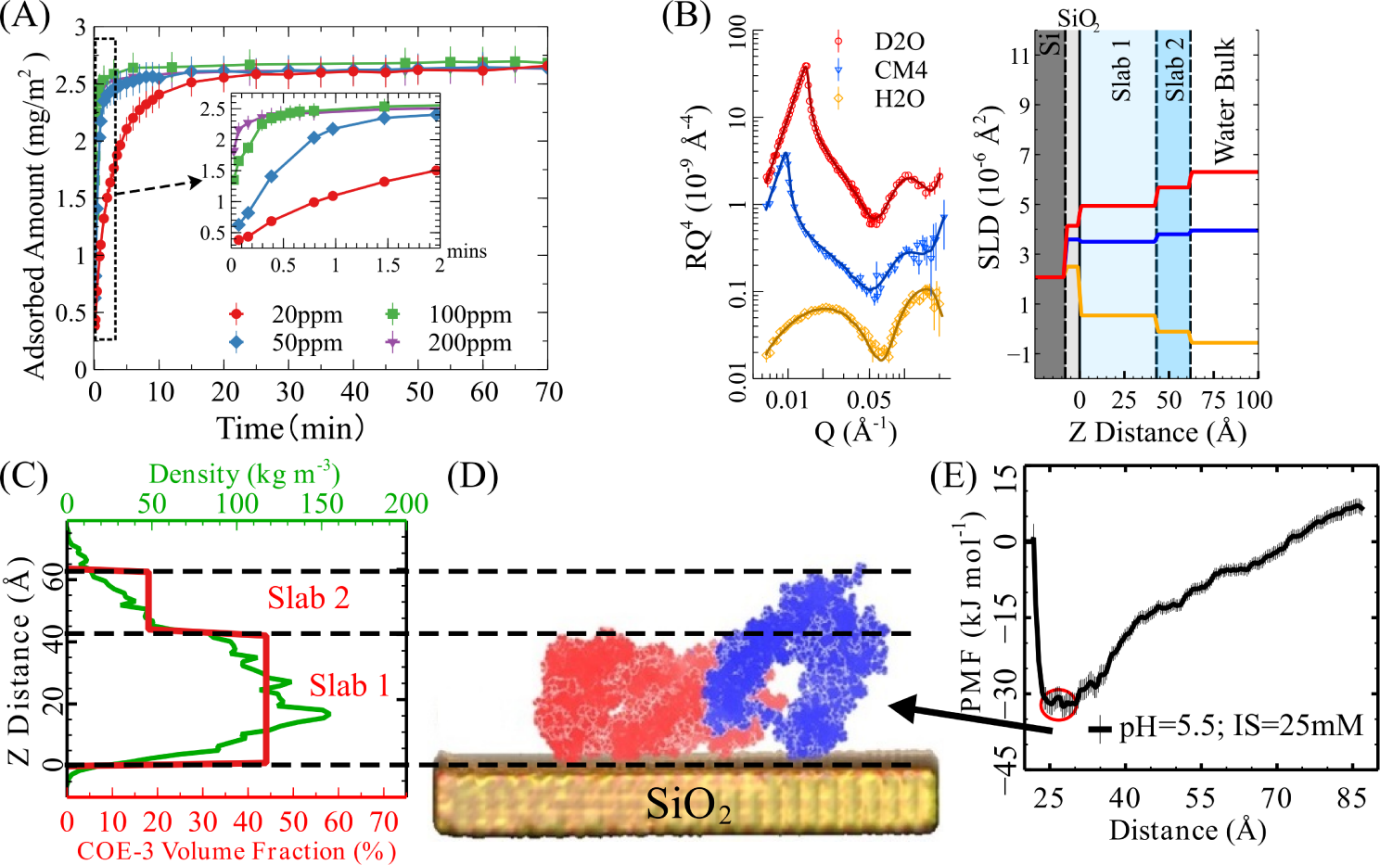
COE-3 adsorption at the SiO_2_/water interface in His buffer (IS = 25 mM, pH 5.5, 20 °C) investigated by SE, NR, and MD. (A) COE-3 adsorption amount over time; the inset shows the initial 2 min of measurement. (B) Left: NR profiles of the equilibrated adsorption layer (without samples in buffer bulk) in D_2_O (red circle), CM4 (blue triangle), and H_2_O (yellow diamond). The best fittings were plotted as solid lines. Profiles for CM4 and H_2_O contrasts were offset by scales of 0.5 and 0.2, respectively, for clarity. Right: Corresponding SLD profiles from best-fit models, with slabs color-coded for clarity. (C) Volume fraction profiles of COE-3 (red line, bottom axis) from NR fitting and density profile from the minimum free energy MD conformation (green line, top axis), plotted against Z distance (the black axis on the left). (D) The minimum free energy MD conformation of adsorbed COE-3 aligns with the optimized NR fitting, with Fc and Fab in blue and red, respectively, and the SiO_2_ surface in yellow. (E) The PMF profile of the COE-3–SiO_2_ interaction showing a free energy minimum when the mAb center of mass is positioned at 27.5 Å from the SiO_2_ surface.

Spectroscopic ellipsometry (SE) was used to investigate the kinetic adsorption process of COE-3 at the SiO_2_/water interface in a His buffer (IS = 25 mM, pH 5.5, 20 °C). Figure 1A shows the amount of COE-3 adsorbed against time at four different concentrations. The results indicate that the amount of adsorbed COE-3 reaches a plateau around 20 min for all concentrations investigated, indicating saturation of the adsorbed COE-3 molecules at the SiO_2_/water interface. Surface saturation occurs when the COE-3 surface density reaches 2.7 ± 0.2 mg/m^2^, even at a bulk concentration as low as 20 ppm (0.02 mg/mL). This suggests that higher concentrations significantly accelerate the adsorption rate, while the saturation amount is relatively independent of bulk concentration. Specifically, higher bulk concentration leads to a shorter time to reach surface saturation due to the higher diffusion rate for material transfer to the surface-closing region and increased interaction rate between the surface and the protein.

To further study the adsorbed layer structure of COE-3 at the surface, we performed NR measurements of the saturated adsorption layer in a D_2_O buffer at bulk concentrations of 20, 50, 100, and 200 ppm. Figure S4A shows that the NR profiles overlap well, indicating that the thickness and density structures of the saturated adsorption layer of COE-3 remain consistent across all concentrations. After rinsing the adsorbed COE-3 layer with the same buffer used for adsorption, we measured the NR profile again. The perfectly overlapped NR profiles in Figure S4B indicate that the equilibrated adsorption layer remained unchanged after rinsing. This suggests that, at pH 5.5, the COE-3 undergoes irreversible deposition rather than achieving dynamic equilibrium.

To gain further insight into the adsorbed layer of COE-3 at surface saturation, we employed NR measurements with three water contrasts: D_2_O, CM4 (contrast-matched water with an SLD of 4 × 10^–6^ Å^–2^), and H_2_O. The left panel of Figure 1B shows the profiles from these measurements, with solid lines representing the best simultaneous fit using a 2-slab model, as detailed in the Materials and Methods section. The SLD profiles resulting from the model fitting, shown in the right panel of Figure 1B were used to calculate the COE-3 volume fraction in each slab. The resulting COE-3 volume fraction profile, plotted against the vertical distance from the SiO_2_/water interface (Z distance), is shown as a red solid line in Figure 1C. Key fitting parameters and the layer structural features derived from this model are given in Table 1. The results show that the adsorbed layer has a total adsorbed amount of 3.14 ± 0.16 mg m^–2^, which is slightly higher than the value obtained from SE measurements. The SLD profiles demonstrate that the adsorption layer comprises two distinct regions with a total thickness of 62.5 ± 2.6 Å. The region closest to the surface has a thickness of 42.5 ± 1.8 Å, containing 84% of the total adsorbed mass. In contrast, the outer, more diffuse region has a thickness of 20.0 ± 1.9 Å, constituting 32% of the total thickness but only 16% of the total adsorbed mass. The mAb volume fraction of this outer region is less than half of the inner region.

**Table 1:** Key Model Fitting Parameters and Structural Features of the COE-3 Adsorbed Layer at Surface Saturation in Buffer pH 5.5 and 9 (IS = 25 mM, 20°C)

*pH*	*Parameters*	Slab 1	Slab 2	Total/Average^a^
*pH 5.5*	*Thickness (Å)*	42.5 ± 1.8	20.0 ± 1.9	62.5 ± 2.6
	*Volume Fraction (%)*	44.1 ± 1.1	18.3 ± 2.8	35.8 ± 1.3
	*Adsorbed Amount*(mg m^–2^)	2.62 ± 0.13	0.51± 0.09	3.14 ± 0.16
*pH 9*	*Thickness (Å)*	35.0 ± 1.3	43.0 ± 2.3	78 ± 2.6
	*Volume Fraction (%)*	40.5 ± 0.8	18.7 ± 0.9	28.3 ± 0.7
	*Adsorbed Amount*(mg m^–2^)	2.00 ± 0.08	1.12 ± 0.08	3.12 ± 0.11

a ″Thickness” and “Adsorbed Amount” values in this column show the sum of values of Slab 1 and Slab 2; the “Volume Fraction” in this column shows the average value of Slab 1 and Slab 2 for volume fraction, weighted by the thickness.

Figure 1E presents the potential of the mean force (PMF) profile of the interaction between COE-3 and the SiO_2_ surface in His buffer (IS = 25 mM, pH 5.5, 20 °C), as calculated through all-atom MD simulations. The PMF profile describes the free energy landscape of the system and reveals a strong, attractive interaction between mAb and the SiO_2_ surface, as evidenced by a clear potential well when the COE-3 approach the surface. The free energy minimum on the PMF profile corresponds to the most stable or preferred state of the protein, with the conformation of COE-3 in its adsorbed state shown in Figure 1D. These MD results provide insight into the specific conformation of each domain of COE-3 during adsorption, extending beyond a simple “flat-on” description. Specifically, the minor axis of the Fabs (approximately 45 Å) is oriented perpendicular to the interface. In comparison, the major axis of the Fc (approximately 70 Å) is nearly perpendicular to the interface, but with a slight tilt. Root mean square deviation (RMSD) analysis further confirms that COE-3 maintains structural stability in its adsorbed state at pH 5.5, with minimal unfolding or denaturation (see SI Section 1.6 and Figure S3).

A density profile was calculated from this simulated COE-3 adsorbed conformation, represented by the solid green line in Figure 1C. For comparison, the volume fraction of COE-3 obtained from the NR measurements is plotted in the same figure by using a solid red line. The two-step function of the volume fraction profile from the NR reasonably aligns with the more detailed density profile obtained from the MD simulation. Moreover, the distance between the mass-center of the adsorbed COE-3 and the SiO_2_ surface is 26.3 ± 1.3 Å from NR measurements and 27.5 Å from MD simulation. Since the volume fraction measured by NR represents an average value along the Z direction for the entire sample, these agreements between experimental and simulation data support the likelihood that most adsorbed COE-3 molecules on the interface adopt the conformation suggested by the MD results.

Furthermore, the MD results explain the volume fraction profile observed by NR. Specifically, the MD simulations show that the inner slab in the NR 2-slab model contains the two entire Fabs and a portion of the Fc, whereas the outer slab only contains the remaining part of the Fc. This difference in the occupancy of the mAb fragments between the inner and outer slabs results in a more diffuse outer slab region.

### pH-Dependent Conformational Change of COE-3: A Reversible Transformation

**Figure 2:**
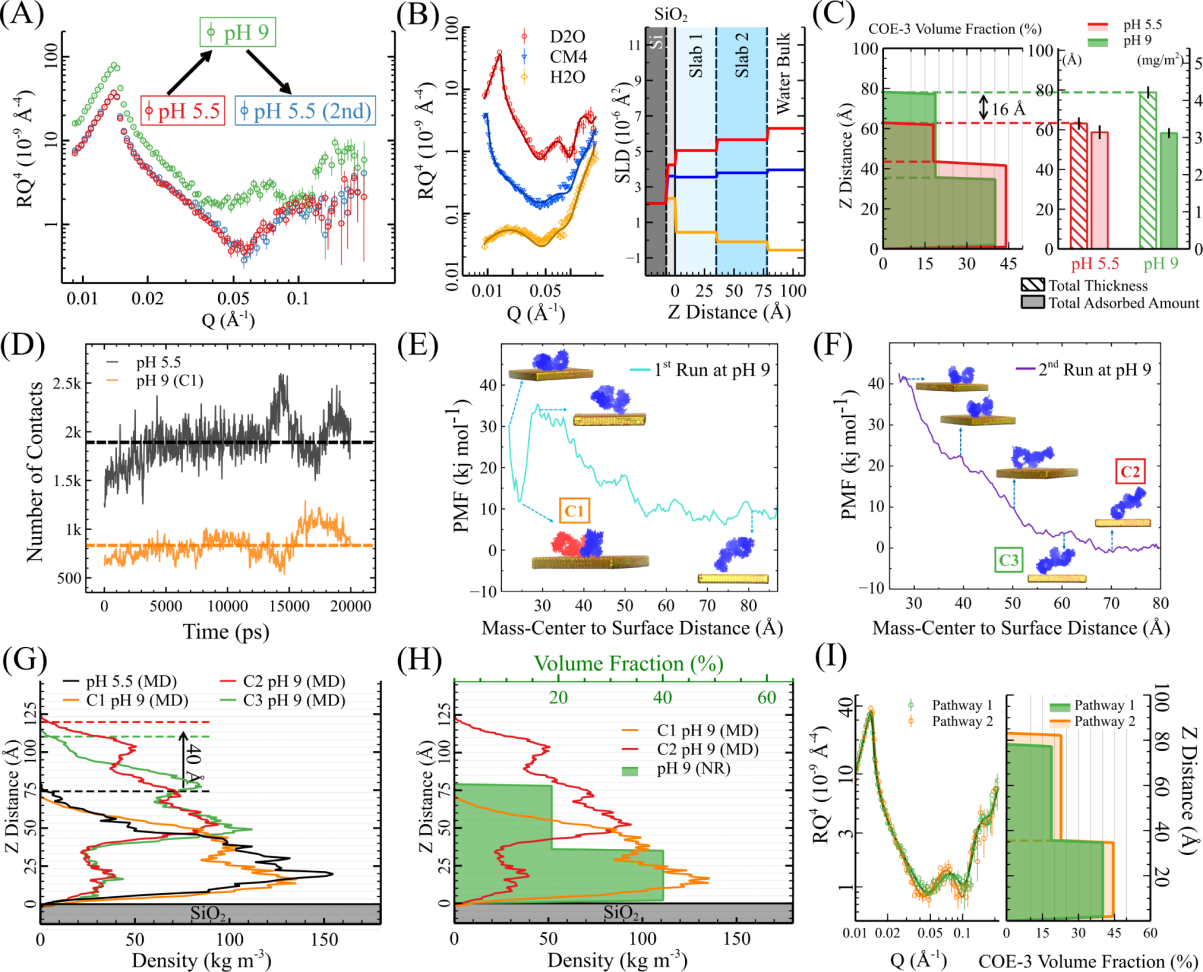
NR analysis revealed pH-dependent reversible conformational changes in adsorbed COE-3 molecules. (A) NR profiles of the surface-saturated COE-3 adsorption layer during a pH cycle: pH 5.5 (red), 9 (green), and back to pH 5.5 (blue). Measurements were conducted on the same sample at each pH through sequential buffer exchange. The NR profile of pH 9 was shifted upward for clarity. (B) Simultaneous fitting of the three NR contrasts of the COE-3 adsorption layer at pH 9. The SLD profiles of the best fitting are shown on the right. (C) Left: the volume fraction profiles of the COE-3 adsorption layer at pH 5.5 (red) and 9 (green), with the dashed line representing the boundary between slab 1 and slab 2. Right: total thickness (striped bar) and adsorbed amount (solid bar) of the COE-3 adsorption layer at pH 5.5 (red) and pH 9 (green). The dashed lines indicate that the total thickness is the sum of the thicknesses of slab 1 and slab 2. (D) The number of atomic contact points between COE-3 and the SiO_2_ surface for the minimum energy conformations at pH 5.5 and 9 (conformation C1). The dashed lines represent the average number of contact points over the time frame for a distance cutoff between SiO_2_ and mAb of 0.5 nm. (E), (F) MD PMF profiles for COE-3–SiO_2_ interactions at pH 9 and IS = 25 mM, obtained from two independent umbrella sampling runs. The corresponding COE-3 conformations at specific positions are labeled accordingly. Figure S5 provides additional protein conformations along the PMF profiles for a comprehensive view, and Figure S6 shows alternative views of conformation C1. For conformation C1, Fab and Fc are distinguished by the colors red and blue. (G) Density profiles of COE-3 conformations obtained from MD simulations at pH 5.5 (black) and pH 9 ((C1: orange, C2: red, C3: green)). (H) Comparison between the MD-derived density profiles of potential COE-3 conformations (solid lines) and the NR-derived volume fraction profiles (green area). (I) Comparison of the COE-3 adsorbed layers at pH 9 formed via two pathways: (green) by first adsorbing COE-3 at pH 5.5, then changing the pH to 9; (orange) by direct adsorption at pH 9.

Following the investigation of the mAb adsorbed layer at pH 5.5, we examined the pH-dependent structural changes in the layer. NR measurements were undertaken while the pH of the sample environment was altered stepwise in a cycle through buffer exchange. As shown in Figure 2A, the NR profile was initially measured at pH 5.5 (red), followed by a measurement at pH 9 (green). The distinct fringes in the profiles reflect variations in the adsorption layer’s thickness with the pH changes. After reverting the pH back to 5.5 via buffer exchange, the NR profile (blue) closely overlapped with the initial profile (red), demonstrating a full recovery of the adsorption layer’s thickness and structure, with no detectable material loss. The reversible pH-dependent thickness and structural changes of the COE-3 adsorption layer arise from the conformational adjustments of adsorbed COE-3.

To better understand the COE-3 conformation at pH 9, we conducted the NR measurements under three solvent contrasts and simultaneously fit the data using a 2-slab model, as illustrated in Figure 2B. The key parameters and deduced structural features are given in Table 1. The volume fraction distributions of the COE-3 adsorbed layer at pH 5.5 and 9 are displayed in the left panel of Figure 2 (C), revealing that the adsorbed layer at pH 9 also divides into two regions: a dense region adjacent to the SiO_2_ surface and a relatively diffuse region above it. The solid-filled bars in the right panel of Figure 2C indicate that the adsorbed amount (surface mass density) remains consistent across pH changes, confirming that little material loss occurred during the pH changes. However, the total thickness of the COE-3 adsorbed layer increases from 62 to 78 Å as the pH rises. The values in Table 1 demonstrate a transformation of material from the dense region to the more diffuse region further from the surface. As a result, the distance from the mass-center to the SiO_2_ surface increased from 26.3 ± 1.3 Å at pH 5.5 to 31.5 ± 1.2 Å at pH 9. These findings suggest conformational changes in the adsorbed COE-3 molecules as pH shifts, leading to the new orientation of the mAb molecules at pH 9, distinct from the “flat-on” conformation at pH 5.5.

To complement the NR data, we conducted MD simulations and performed multiple independent umbrella sampling runs. The PMF profiles of mAb-SiO_2_ interactions at pH 9, as shown in Figure 2E,F, exhibit two distinct features. Initially, both runs show a significant decrease in free energy with distance, indicating a repulsion between the protein and the surface. Beyond 55 Å from the substrate, the PMF curves level off, reaching a stable region of the system’s free energy. This plateau suggests the presence of multiple conformations at similar energy levels and highlights the flexibility of the mAb in adjusting its conformation relative to the surface. Interestingly, in the first run, we observe a local energy minimum at 25 Å from the surface (Figure 2E), with a free energy comparable to the stable energy state in the plateau region. We note, however, that sampling the local minimum requires crossing a high activation barrier (>20 kJ/mol). This event can take place in experiments, but it is unlikely to take place spontaneously using standard MD simulations, hence the need to employ a biasing technique as we have done here.

To represent the system at pH 9, we select three representative COE-3 conformations at the following minima: C1 (from the first run at the local minimum) and C2/C3 (from the second run at points in the plateau region). An RMSD analysis was conducted at pH 5.5 and 9 to confirm structural stability across these conformational shifts. This analysis, detailed in SI Section 1.6, shows minimal structural deviations, confirming that pH changes do not lead to significant unfolding or denaturation of COE-3 (see Figure S3). Figure 2G presents the density profiles of these conformations, along with the minimum energy conformations at pH 5.5 (discussed in mAb and Fragments Samples Section). Notably, C1 has a mass distribution comparable to the “flat-on” conformation at pH 5.5. However, the reduced number of atomic contact points with the surface of C1, defined as the number of mAb atoms within 0.5 nm of the silica surface (Figure 2D), suggests a looser interaction with the interface. This observation is further supported by the visual flexibility in C1 (see Figure S6 for alternate views), where the fragments are more flexibly oriented, reflecting a reduced degree of surface attachment compared to the more stable interaction seen at pH 5.5. Conformations C2 and C3 exhibit a further mass away from the surface (Figure 2G), with the maximum distance between the molecule and the surface increasing by approximately 40 Å compared with minimum energy conformations at pH 5.5. The conformational schematic in Figure 2F, showing the mAb fragments positioned further from the surface, suggests a tilted orientation that reflects a more flexible and loosely attached state at pH 9, even more so than C1.

Figure 2H compares the density profiles of selected conformations (C1 and C2) with the volume fraction profile of the COE-3 adsorption layer at pH 9. This comparison clearly shows that conformation C1 more closely matches the experimental data, suggesting that it is the more prevalent mAb conformation under these conditions. However, the experimental data indicate a mass-center-to-surface distance of 32.7 Å, greater than the value for C1 (approximately 25 Å). This discrepancy arises because the volume fraction profile obtained from NR represents an average across all adsorbed materials within the measured area. Therefore, the observed mass-center reflects a combination of coexisting conformations at the interface. While C1 is the dominant conformation, the presence of other more flexible conformations, such as C2 and C3, also contributes, shifting the mass further away from the surface and increasing the overall adsorption layer thickness.

Building on our analysis of COE-3 conformations across pH levels, we further investigated how the pathway of adsorption at pH 9 influences the resultant adsorbed layer structure and density. We examined the adsorption layers of COE-3 at pH 9 formed through two pathways. The first pathway, as mentioned earlier, involves the initial adsorption in a pH 5.5 buffer (IS = 25 mM) followed by a subsequent change in pH to 9. The second pathway involved preparing the COE-3 sample in a pH 9 buffer (IS = 25 mM) and applying it to the bare SiO_2_ surface for adsorption. Figure 2I illustrates the NR and material volume fraction profiles derived from the best-fit models. Remarkably, the adsorption layers formed at pH 9 through both pathways exhibit similar thickness and structure, suggesting that the molecular conformation of COE-3 adopted at pH 9, after the pH cycle (from 5.5 to 9), closely resembles its preferred conformation during direct adsorption at pH 9. However, the adsorption layer directly formed at pH 9 exhibits a higher material volume fraction in both the dense and diffuse regions than in the first pathway, leading to a 20% greater total adsorption amount. This disparity can be attributed to the lower averaged molecular footprint and fewer attached sites of COE-3 at pH 9 than at pH 5.5. Also, from the perspective of overall net charge, as the pH approaches the mAb’s overall pI (8.44), protein–protein repulsion is reduced. Consequently, a higher packing density of molecules is allowed, leading to the observed increase in the total adsorbed amount.

### Adsorption of Fab and Fc Fragments

To better understand the pH-dependent conformational changes of mAbs, we investigated the adsorption behavior of the Fab and Fc fragments separately using NR. Following the same procedure as that for COE-3, the fragment samples were first applied to the SiO_2_ surface in His buffer (IS = 25 Mm, pH 5.5). After the maximum adsorption was reached, the solution was replaced by buffer exchange. NR measurements under 3 solvent contrasts and simultaneous fitting were conducted, as shown in Figure S7. For both Fab and Fc adsorbed layers, a one-slab model was sufficient to fit the NR results.

**Figure 3:**
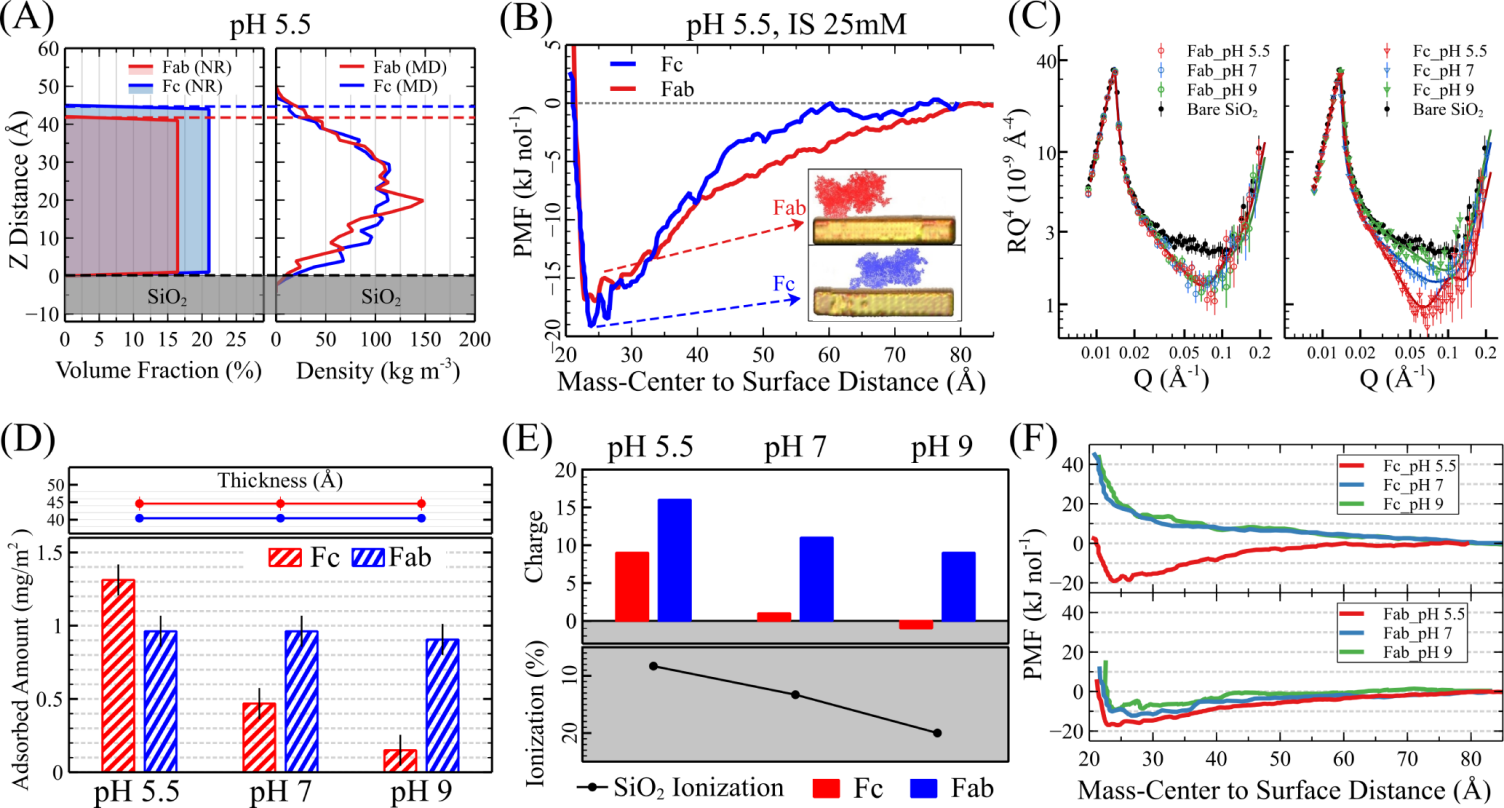
Characterization of adsorption of COE-3′s Fab and Fc fragments at the SiO_2_/water interface. (A) Material volume fraction (left) and density profiles (right) for Fab (red) and Fc (blue) at pH 5.5, obtained from the NR results and MD simulations. (B) PMF profiles for the interaction between the SiO_2_ surface and Fab (red) and Fc (blue) at pH 5.5, with minimum free energy conformations shown. (C) NR profiles for Fab (circles) and Fc (triangles) adsorption at pH 5.5 (red), pH 7 (blue), and pH 9 (green), with best-fit curves; a profile for the bare SiO_2_/buffer interface is included for comparison. (D) Thicknesses and adsorption amounts for Fab and Fc at different pH values were obtained from NR data analysis. (E) Total molecular charges of Fc (red) and Fab (blue) fragments across pHs, with SiO_2_ ionization percentage shown as black points and trend line, which were obtained from the MD simulation. Note that the gray background highlights that SiO_2_ remains negatively charged across the entire pH range. (F) Simulated PMF profiles showing the interaction between the SiO_2_ surface and Fc fragment (top) and Fab fragment (bottom) at pH 5.5, 7, and 9.

The material volume fraction profiles for Fab and Fc adsorbed layers are displayed in Figure 3A (left), with thicknesses and adsorbed amounts presented in Figure 3D. At pH 5.5, both fragments show similar adsorption behavior. The thicknesses of the adsorbed layers were close, with 41 Å for Fab and 44 Å for Fc. These values suggest that both adsorbed layers could be modeled by a monolayer of Fab or Fc molecules, with the fragments’ short axis perpendicular to the SiO_2_ surface. The results of the MD simulation confirmed this, as the PMF profiles shown in Figure 3B indicated that the minimum free energy occurs at around 25 Å between the center of mass of each fragment and the surface. The corresponding molecules’ conformations at free energy minima are displayed, and the density profiles calculated from the conformations are plotted in Figure 3A (right). The almost symmetric density profiles confirmed the consistency between the experiments and simulation.

To investigate the pH-dependent behavior of the Fab and Fc, we conducted additional NR measurements while gradually changing the environment pH from 5.5 to 9. The measured NR profiles are displayed in Figure 3C, with the corresponding thicknesses and adsorption amounts, derived from the best-fit models, presented in Figure 3D. Based on the NR signals relative to the bare SiO_2_ and the fitted numerical results, we concluded that the Fab adsorption layer remained stable with little change, while the Fc desorbed progressively as the pH increased. At pH 9, most Fc molecules had desorbed from the surface, leaving only a small amount of material remaining. Although the adsorption amount of the Fab decreased with increasing pH, the layer thickness remained unchanged, indicating no alteration in Fab’s conformation and orientation during desorption. This decrease in the adsorbed amount is attributed to the progressive reduction in the number of intact Fab molecules on the surface as the pH increases.

These phenomena can be discussed within a simplified framework of fragment pI and net charge to approximate the electrostatic interactions. At pH 5.5, both Fab and Fc have positive net surface charges (Figure 3E), while SiO_2_ is negatively charged, resulting in strong electrostatic attraction driving adsorption. However, as the pH increases, the net positive charge on the Fc surface decreases, approaching neutrality at pH 7 and becoming slightly negative at pH 9 (pI of Fc ∼ 6.36). This results in a shift from attractive to repulsive interactions, leading to Fc desorption. In contrast, Fab retains a positive charge even at pH 9 (pI of Fab ∼9.64), allowing it to remain adsorbed to the increasingly negatively charged SiO_2_ surface. The PMF profiles from MD shown in Figure 3F provide further clarity on this finding. The Fab PMF profiles show a consistent energy minimum position across all pH levels, indicating a stable adsorbed state regardless of pH. In contrast, the Fc PMF profiles show significant changes between pH 5.5 and higher pH levels (pH 7 and 9). At higher pH levels, the Fc PMF profiles exhibit a continuous decrease in energy with an increasing distance from the surface, indicating the absence of an energy-stable adsorbed position.

Thus, both NR experiments and MD simulations demonstrate that at pH levels (pH 7 and 9) between pI values of Fc and Fab, the Fc begins to desorb, while the Fab remains adsorbed. This finding explains the conformational changes observed in adsorbed COE-3 as the pH varies. At pH 5.5, which is lower than the pI of either fragment, all fragments attach to the surface, favoring a “flat-on” conformation. As the pH is increased to 9, Fab remains attracted to the surface, keeping the entire antibody attached, while the Fc fragments are repelled by the surface, leading to the “tilted” conformation of the entire mAb.

## Conclusion

This study provides a comprehensive understanding of the pH-dependent conformational behavior of COE-3 at the SiO_2_/water interface using a combination of NR and MD simulations. To simplify the complex electrostatic interactions captured by MD, we interpret these behaviors within a dual-pI framework considering the distinct pI values of the Fab and Fc fragments. At pH 5.5, which lies below the pIs of both Fab and Fc fragments, COE-3 undergoes irreversible adsorption in a stable “flat-on” orientation, where all fragments are attracted to the surface. However, at pH 9, between the pIs of the Fab and Fc, the antibody exhibits a reversible shift toward “tilted” orientations and more flexible conformations, with fewer attachment points to the surface. This shift is driven by differential electrostatic interactions of the fragments: while the Fab, with a higher pI, remains firmly attached to the surface, the Fc fragment desorbs, repelled by the surface’s increasingly negative charge. This dual-pI framework may provide a novel approach for the rational design of mAbs, where fine-tuning the pI positions of Fab and Fc could enable precise control over the mAb conformation and orientation at target pH levels. Unlike the conventional framework using mAb’s overall pI, this dual-pI approach better accommodates fragment-specific behavior.

Kanthe et al.^41^ reported that at the air/water interface, mAbs exhibit orientation changes with increasing concentration, shifting from a “flat-on” to a “tilted” orientation primarily due to crowding or enhanced lateral protein–protein interactions. This transition is accompanied by partial unfolding with hydrophobic interactions at the interface reconfiguring parts of the structure into β-sheets. In contrast, at the SiO_2_/water interface, we observe orientational changes in COE-3 driven by distinct mechanisms. Our RMSD analysis confirms that COE-3 retains structural stability across pH adjustments, with minimal unfolding or denaturation. The pH cycle pathway experiments demonstrate that the orientational shifts at pH 9 are not crowding-driven, as there is no gain in the adsorbed amount. Additionally, the direct adsorption pathway at pH 9 shows a higher adsorption amount than at pH 5.5, suggesting that lateral protein–protein interactions are likely weaker at pH 9. Given that the orientation shift occurs during the pH transition from 5.5 to 9, this indicates that the shift is not caused by enhanced protein–protein interactions.

However, further investigation is warranted to clarify the role of protein–protein interactions and potential spatial heterogeneity along the surface. In this study, MD simulations focused on individual mAb monomers; incorporating multiple monomers in future simulations could offer insights into potential clustering and the effects of protein–protein interactions, particularly at elevated concentrations. Moreover, NR measurements provide an averaged volume fraction profile of the COE-3 adsorbed layer and cannot directly capture the spatial heterogeneity. While the absence of off-specular reflection in our NR data suggests no significant clustering or roughness, this does not entirely exclude the possibility of mild heterogeneity in the adsorption pattern. Another key challenge lies in translating these findings to real-world biosensors or therapeutic applications. In practical settings, factors such as fluctuating pH, complex biological media, and mechanical stresses may impact mAb stability and conformation differently than in controlled laboratory environments.^42,43^

In summary, this study advances our understanding of the pH-dependent conformational plasticity of mAbs at the solid–liquid interface, particularly in relation to fragment-specific interactions with a charged SiO_2_ surface. Our findings enrich the theoretical framework of mAb adsorption and open up innovative possibilities for optimizing mAb-based biosensors through precise control of orientation and conformation at the interface. In addition, the findings address a critical knowledge gap in understanding mAb interfacial behavior and could lead to strategies for protein adsorption prevention and mitigating protein aggregation. Finally, our analysis strategy of combining NR and MD establishes a framework and serves as a pioneering example for future research into protein interfacial behavior.

## Materials and Methods

### mAb and Fragment Samples

The human IgG1κ antibody, denoted as COE-3, was provided by AstraZeneca as a stock solution at 46.4 mg/mL in 25 mM histidine/histidine hydrochloride (“His buffer”), 7% w/v sucrose, pH 6.0. The COE-3 mAb was expressed in Chinese hamster ovary cells, purified, and concentrated using industry-standard methods. The COE-3 has a MW of 144.8 kDa and contains two identical Fabs and one Fc. Each Fab contains 442 amino acid residues and has a MW of 47.3 kDa; the Fc (including the hinge region) contains 446 amino acid residues and has a MW of 50 kDa. The Fc and Fab fractions were obtained through a COE-3 cleavage process by papain digestion and a separation and purification process by chromatography. The sequence information has been published in a previous study.^29^ All mAb and fragment samples were stored at −80 °C and thawed before being dialyzed and diluted in the appropriate buffer solution for experiments. The buffer, formulated as described in SI Section 1, was prepared using histidine and histidine hydrochloride for pH 5.5, and phosphate salts for pH 7, 8, and 9. The formulations were replicated for the D_2_O buffers, which were used in neutron experiments. Buffer pHs, confirmed postpreparation within an error range of ±0.1, required no titration due to their precise formulation calculations.

### Silicon Wafer and Blocks

Silicon wafers (⟨111⟩ orientation, minimal doping) with one-side optically flat were purchased from Compart Technology, UK. The wafers were cut into 2 × 2 cm^2^ to fit the solid/liquid cell specially built for spectroscopic ellipsometry measurements. The silicon blocks (⟨111⟩ orientation, 5 × 8 × 2 cm^3^) were one-side polished into an optically flat surface by Crystran Ltd. (Poole, UK). The optically flat silicon wafer and block surfaces bear an ultrathin native oxide layer. All the silicon oxide surfaces were cleaned before use following an RCA standard clean-1 procedure.^44^ A 5% (w/w) Decon 90 solution (Decon Ltd., UK) was used to remove the adsorbed mAb molecules and regenerate the silicon oxide surface between measurements.^45^

### Chemicals

D_2_O (99% D), sodium chloride, histidine, histidine hydrochloride, disodium hydrogen phosphate, and sodium phosphate monobasic for buffer preparation were purchased from Sigma-Aldrich. The pure H_2_O was processed from a Millipore UHQ system at 18.2 MΩ·cm (Merck-Millipore, Watford, U.K.).

### Spectroscopic Ellipsometry (SE)

The kinetic adsorption was investigated by using a Woollam spectroscopic ellipsometer M2000 (J.A. Woollam Co. Inc.) over a wavelength range between 200 and 800 nm.^27,28,46^ Ellipsometry quantitatively investigates light polarization change upon reflection in the form of the complex reflectance ratio by measuring the wave amplitude ratio and the wave phase difference. The complex reflectance ratio is a function of the wavelength λ. Also, it can be calculated theoretically by using an interfacial structure model containing homogeneous and isotropic layers described by thickness and refractive index parameters. The thickness and Cauchy’s coefficients of the adsorbed protein layer were determined through an iterative fitting process using the software developed by J.A. Woollam Co. Inc. This work used a specially designed liquid cell to achieve measurements at the solid/liquid interface. The thicknesses of the native oxide surface layers of the wafers were characterized before the experiment. The surface density of the adsorbed protein layer was calculated using De Feijter’s formula^47^ (eq 1)),
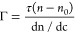
1where *τ* is the thickness of the adsorbed layer, **n*_0_ is the refractive index of ambient buffer at 630 nm and dn/dc is the refractive index concentration increment of protein at 630 nm, which was taken as 0.18 mL/g for proteins in this work. Cauchy’s equation described the refractive index of the adsorbed protein layer with Cauchy’s coefficients (*A*, *B*) equal to 1.45 and 0.003, respectively. Although the thickness and refractive index coupling issues remained challenging, the surface density as the product of these two parameters ensured good consistency and reliability in the analysis.*

### Neutron Reflection (NR)

The details of NR theory and application related to protein adsorption can be found elsewhere.^48−50^ In short, the NR measures the neutron SLD profile at the interfaces in the direction (Z axis) perpendicular to the interface. This technique is particularly useful for studying key structural parameters such as the composition, thickness, and roughness of thin interfacial layers. When dealing with the interfacial adsorption of a single material with a homogeneous distribution of SLD, the SLD profile at the interface can be directly converted to the material’s density distribution at the interface. NR measurements in this work followed the method in the previous work^27,28^ and were undertaken on the POLREF and INTER reflectometers at ISIS Neutron Facility, Rutherford Appleton Laboratory, UK.^51,52^ The prepared silicon block was placed within a cell designed for solid–liquid interfaces and mounted on a sample stage. A highly collimated neutron beam was directed at the surface of the silicon block, and a detector measured the intensity of the reflected neutron beam from the surface. The reflectivity varied with the momentum transfer (*Q*) of the neutrons, which was determined from the incident angle (θ) and the wavelength of the neutrons (λ) as *Q* = (4π sin θ)/λ. The solid–liquid flow cell was connected to an HPLC pump, which managed alterations in the bulk solution to attain various buffer conditions and isotopic contrasts. In this study, an appropriate mixture of H_2_O (SLD = −0.56 × 10^–6^ Å^–2^) and D_2_O (SLD = 6.35 × 10^–6^ Å^–2^) was used as the buffer solvent to achieve isotopic contrast variation. The substrates were initially characterized with two contrasts of water (H_2_O, D_2_O) before exposure to the sample solutions. The mAb adsorption layers were characterized with three contrasts of water (H_2_O, D_2_O and Contrast Matching SLD = 4 (CM4) water). All the measurements in this study were performed at room temperature (20 ± 1 °C).

The initial reflectivity data reduction was processed using Mantid software.^53^ The data were then analyzed using a multiple-slabs model with the Motofit fitting package^54^ and RasCAL fitting package,^55^ which uses the NR theory and the optical matrix method to simulate reflectivity profiles. A least-squares minimization is utilized to minimize the discrepancies between the modeled and experimental data. To determine the uncertainty in the model parameters, we performed a bootstrap analysis with 1000 resamples using the RasCAL program.^56^

The multiple-slab model was used with the minimum number of slabs required to achieve a satisfactory fitting level. An Elbow Plot of the normalized Chi-square values shown in Figure S8 indicates that a 2-slab model provided the best balance between fit quality and model complexity for both pH 5.5 and pH 9. Each slab was defined by its thickness and SLD in the multiple-slabs model. The SLD of a slab can be calculated by considering the contributions from the water bulk and the mAb, as described in eq 2:
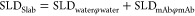
2where φ_water_ and φ_mAb_ are the volume fractions, and the sum of the φ_water_ and φ_mAb_ must be unity. The total adsorbed amount (Γ) can be determined using eq 3:
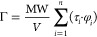
3where τ_*i*_ and φ_*i*_ are the thickness and protein volume fraction of the slab *i*, respectively; *n* is the number of slabs used in the multiple-slabs model; MW and *V* are the molecular weight and volume of the protein, respectively.

The key parameters used in eq 3, together with the SLDs required for performing the analysis using eq 2, are presented in Table 2. The SLDs of Fab, Fc, and COE-3 fluctuate under different H_2_O/D_2_O mixtures due to the transfer of labile hydrogen on the amide bond linkages and polar and charged side chains with deuterium from the solvent.

**Table 2:** Key Physical Parameters Used for the Model Fitting of Neutron Reflectivity, where MW, *V*, SL, and SLD Denote Molecular Weight, Volume, Scattering Length, and Scattering Length Density, Respectively

Component	MW (g mol^–1^)	V (Å^3^)	Contrast	SL (10^–5^ Å)	SLD (10^–6^ Å^–2^)
COE-03	144 827	171 740	H_2_O	33 266	1.94
			CM4	49 358	2.88
			D_2_O	57 648	3.36
Fc	49 970	59 664	H_2_O	11 374	1.906
			CM4	16 673	2.79
			D_2_O	19 403	3.25
Fab	47 429	56 038	H_2_O	10 946	1.95
			CM4	16 343	2.91
			D_2_O	19 123	3.41

### Molecular Dynamics (MD)

MD simulations were performed to calculate the potential of mean force (PMF) profiles for the interaction between COE3/Fab/Fc and the silica surface, using the umbrella sampling technique.^57^ The component along the normal to the silica surface of the distance between the centers of mass of the silica slab and the proteins was used as the reaction coordinate (ξ; see Figure S2 of SI Section 1 for details). All the simulations were performed using the Gromacs (2021.3) simulation package. The Charmm36m force field (ff)^58^ (with the TIPs3P water model) was used for the proteins, and the INTERFACE ff^59^ was used to describe the silica slab. A short-range cutoff of 1.2 nm was used for the dispersion interactions. This cutoff is customary in simulations due to its efficiency in computational time. However, we introduced corrections for both energy and pressure to include the full range of the dispersion interactions. The LINCS algorithm was used to restrain all bonds involving hydrogens, and the Particle Mesh Ewald method was used to evaluate the electrostatic interactions. A time step of 2 fs was used for the MD runs. The details of the procedure used to build the initial system and the composition of systems at different pHs are given in SI Section 1 (Tables S1, Figure S1 and the associated text). The charges of the proteins and silicon surface at different pHs are listed in Tables S2, S3. The systems built with the protein placed on the silicon surface in an aqueous environment in the presence of buffer and salt (see Table S1) were initially minimized and then subjected to a short 2 ns equilibration with the Berendsen barostat^60^ and v-rescale thermostat^61^ for pressure and temperature coupling, both with coupling constants of 0.5 ps. This was followed by a 5 ns long production run performed in the NPT ensemble with the Parrinello–Rahman barostat^62^ (coupling constant of 2.0 ps) and the v-rescale thermostat (coupling constant of 0.5 ps).

Next, a nonequilibrium pulling simulation was performed to separate the protein from the silica surface at a pulling velocity of 0.005 nm ps^–1^. From the pulling trajectory, 65 configurations were extracted with ξ ranging from 3.1 to 9.5 nm at an interval of 0.1 nm. These 65 configurations were the starting points for 65 parallel umbrella window simulations with ξ restrained to the initial values using a harmonic restraint of 1000 kJ/mol/nm^2^. The systems were equilibrated in each window for 5 ns (Berendsen barostat with a coupling constant of 0.5 ps and a v-rescale thermostat with a coupling constant of 0.5 ps). After equilibration, a minimum of 30 ns long production runs were performed in each window by employing the Parrinello–Rahman barostat with a pressure coupling constant of 2.0 ps. This allowed for data collection across a range of distances between the protein and the silica surfaces. Using histograms of the distance/forces between the silica slab and protein for different windows, a PMF profile was generated using the umbrella sampling technique. The PMF profiles were calculated using the weighted histogram analysis method (WHAM)^63^ as a function of ξ. Because the thickness of the SiO_2_ slab was set to 2 nm, the distance between the center of mass of the protein (Fab/Fc/COE-3) and the SiO_2_ surface along a direction normal to the plane equals (ξ – 1) nm. Root mean square deviation (RMSD) calculations were performed on the COE-3 mAb at pH 5.5 and pH 9, both in bulk and in contact with the SiO_2_ surface, of which details are given in SI Section 1.6.

## Supporting Information Available

Descriptions and additional results of the MD simulations for the adsorption of Fab, Fc, and mAb at the solid/water interfaces, RMSD results, and the additional fitting to the neutron reflectivity profiles (PDF)
