# Supplementary material for: pH-Dependent Conformational Plasticity of Monoclonal Antibodies at the SiO2/Water Interface: Insights from Neutron Reflectivity and Molecular Dynamics
Source: ACS Appl Mater Interfaces. 2024 Dec 12;16(51):70231–41. doi: 10.1021/acsami.4c14407 (PMC11672230; doi:10.1021/acsami.4c14407)
Supplement: Supplementary file 1 — am4c14407_si_001.pdf [file am4c14407_si_001.pdf]

Supporting Information

pH-Dependent Conformational Plasticity of Monoclonal Antibodies at SiO_2/Water Interface: Insights from Neutron Reflectivity and Molecular Dynamics

Zongyi Li[1], Suman Saurabh[2], Peter Hollowell[1], Cavan K. Kalonia[3], Thomas A. Waigh[1], Peixun Li[4], John R. P. Webster[4], John M. Seddon[2], Fernando Bresme[2], Jian Ren Lu[1]*

1 Biological Physics Group, School of Physics and Astronomy, Faculty of Science and Engineering, Oxford Road, The University of Manchester, Manchester, M13 9PL, United Kingdom

2 Department of Chemistry, Molecular Sciences Research Hub Imperial College, London, W12 0BZ, United Kingdom

3 Dosage Form Design and Development, BioPharmaceutical Development, BioPharmaceuticals R&D, AstraZeneca, Gaithersburg, Maryland 20878, United States

4 STFC ISIS Facility, Rutherford Appleton Laboratory, Didcot, OX11 0QX, United Kingdom

*To whom all correspondence should be made. Email: j.lu@manchester.ac.uk

SI 1. Molecular Dynamics (MD) Simulation

1. Building Initial Systems

The initial systems were generated using CHARMM-GUI[1]. To match the experimental conditions, we built the systems corresponding to pH 5.5, 7 and 9. We used the histidine buffer for the systems corresponding to pH=5.5; for pH=7 and 9, we used the phosphate buffer. Na^+ and Cl^- ions at different concentrations were used as the excipient. A total of 10 systems were built. A list of systems is provided in **Table S1** below:

Table S1. List of systems simulated in this work.

S.no	System name	HIS^0 Conc. (mM)	HIS^+ Conc. (mM)	NaCl Conc. (mM)	Ph^{2-} Conc. (mM)	Ph^{1-} Conc. (mM)	System size (no. of atoms)
1.	$Fc^{5.5}_{25}$	5.25	25	0	-	-	706520
2.	$Fc^{5.5}_{200}$	4.82	25	175	-	-	695424
3.	Fc^{7}_{25}	-	-	0	6.25	6.26	707668
4.	Fc^{9}_{25}	-	-	0	8.31	0.08	708046
5.	$Fab^{5.5}_{25}$	5.25	25	0	-	-	706459
6.	$Fab^{5.5}_{200}$	4.82	25	175	-	-	695439
7.	Fab^{7}_{25}	-	-	0	6.25	6.26	707843
8.	Fab^{9}_{25}	-	-	0	8.31	0.08	707976
9.	$COE\text{-}3^{5.5}_{25}$	5.25	25	0	-	-	1107199
10.	$COE\text{-}3^{9}_{25}$	-	-	0	8.31	0.08	1109650

The concentrations of different histidine (His^0 and His^+) and phosphate (Ph^{2-} and Ph^{1-}) charge states were set to match the experimental values. The system sizes range from close to 700,000 (for the Fab and Fc fragments) to 1.1 million atoms (for COE-3).

1.1. Protein charge

The charges of the proteins at pH = 5.5, 7 and 9 were determined using the propKa3.1 methodology[2]. The charge of the Fab domain varies from +16e to +9e as pH increases from pH 5.5 to 9, with some His and Glu residues becoming protonated at lower pH values. Similarly, the charges of Fc were found to be -1e at pH 9 and reached a value of +9e at pH 5.5. The net charges of COE-3 varied from +15e to +40e as pH changed from 9 to 5.5, but these values were not equal to the net sums of the Fab and Fc charges at respective pH values, because the chemical

environments of the amino acids near the hinge of COE-3, differed from that for the same amino acids in the free Fab and Fc domains. The charges of Fc, Fab and COE-3 for different pH values are listed in **Table S2**:

Table S2. Charges of the Fab, Fc and COE-3 at different pH values.

pH	Protein		
	Fc	Fab	COE-3
5.5	+9	+16	+40
7	+1	+11	-
9	-1	+9	+15

1.2. CHARMM-GUI Inputs

Silica slab of area 15 nm × 15 nm for the Fab and Fc systems and 21 nm × 21 nm for the COE-3 system was generated using the nanomaterial modeller module of Charmm-GUI [3]. The thickness of the slab was set to 2 nm. The protonation state of the slab varies with pH. The percentage ionization and surface charge density of the silica surface corresponding to the three pH values employed in our simulations is listed in **Table S3**.

Table S3. % ionization of the silica surface at different pH values.

pH	% Ionization	Surface Charge Density (e/nm^2)
5.5	8.3	-0.8
7	13.3	-1.25
9	20	-1.92

1.3. Protein and buffer

The input-generator module of Charmm-GUI [4] was used to prepare the Fab, Fc and COE-3 proteins with appropriate charges (corresponding to the pH values of 5.5, 7 and 9). The charges were fixed by fixing the protonation states of amino acid residues based on the propKa [2] calculation. The input-generator module was again used to generate initial parameter files for the positively charged and neutral histidine zwitterions and the phosphate ions with charges -2e and -1e. The concentration of different charge states of the buffer molecules is listed in **Table S1**.

1.4. Building the initial systems for simulation

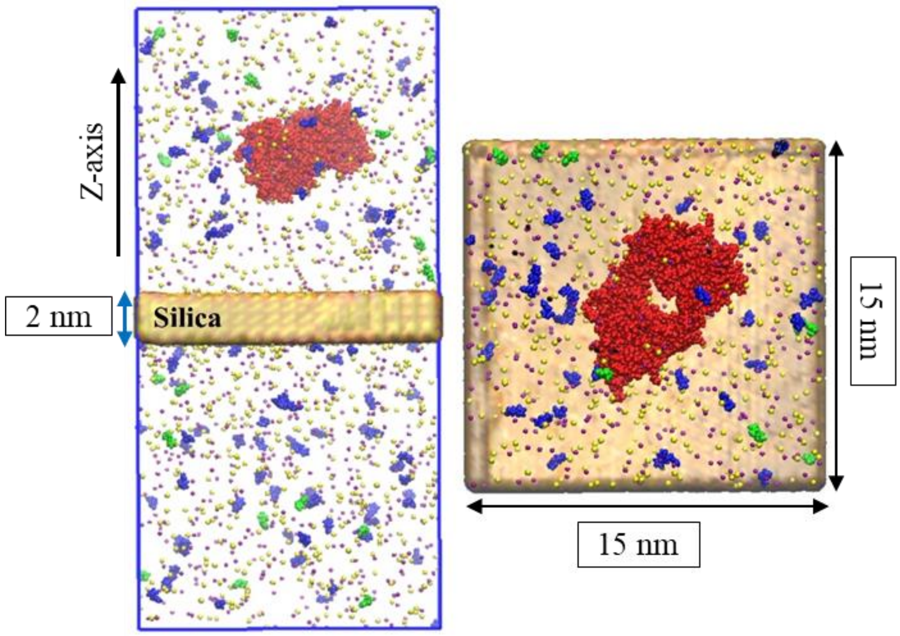

Figure S1. The initial system at pH 5.5. The molecule colored in red is the Fab domain. The molecules colored in blue and green are the positively charged and neutral histidines, respectively. The yellow and purple beads are the Na^+ and Cl^- ions. The top-view of the system is shown on the right.

Four different systems were built for Fab and Fc domains. Two systems correspond to a pH of 5.5 with total ionic strengths of 25 mM and 200 mM, one each corresponding to pH values of 7 and 9 and an ionic strength of 25 mM. For COE-3, two systems were built, one corresponding to pH 5.5 and the other to pH 9, both with an ionic strength of 25 mM. The *Multicomponent assembler* module of Charmm-GUI [5] was used to put together the different components of the systems based on the compositions shown in **Table S1**. As an example, the initial setup for the Fab domain at pH 5.5 is illustrated in **Figure S1**. The proteins were initially placed flat-on with their center of mass close to 3.5 nm away from the central plane of the slab along the Z-direction, which placed them in contact with the silica surface.

1.5. Reaction-coordinates

For Fab and Fc systems, the reaction coordinate was the component of the distance between the centers of mass of the silica slab and the protein (Fab/Fc) along a direction perpendicular to the silica surface.

For COE-3, the reaction coordinate was the distance between the center of mass of the silica slab and the center of mass of the inner ring of domains of the mAb around the hinge (see **Figure S2**).

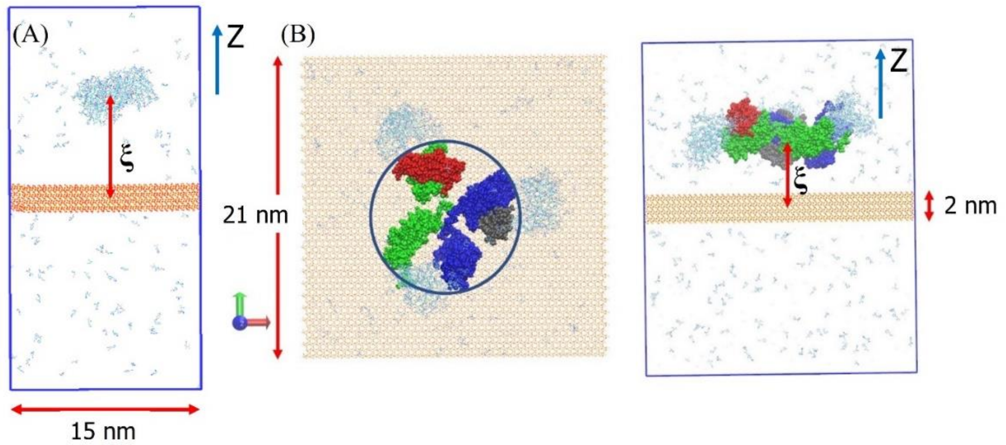

Figure S2. The reaction coordinate ξ for the Fab/Fc systems (A) and the COE-3 system (B) of the top view (left) and side view (right).

The free energy calculation methodology is discussed in the main text.

1.6. Root mean square deviation (RMSD) Analysis

To assess the structural stability of COE-3 under different pH conditions and surface interactions, we calculated the RMSD for each fragment (Fab1, Fab2, Fc) at pH 5.5 and pH 9, both in bulk and in contact with the SiO_2 surface. The RMSD values were calculated relative to the same initial reference conformation in bulk water, with a distance between the hinge and the SiO_2 surface of ~ 5 nm.

For the surface calculations and pH = 5.5, we used the trajectory for the protein-surface distance corresponding to the minimum in the PMF, represented in **Figure 1 panel E** of the main paper. For pH = 9, the analysis was conducted using the trajectory corresponding to the minimum in C1, shown in **Figure 2 panel E** of the main paper.

As shown in **Figure S3**, the very similar results for the RMSD of the bulk systems at pH 5.5 and 9 indicate that the pH does not lead to a significant change in the fragment structure. For the proteins at the surface, there are slight differences between the Fab1 RMSDs, but these are small. Therefore, we conclude that the pH and the interaction with the surface do not induce protein denaturation or lead to significant structural changes in the protein fragments.

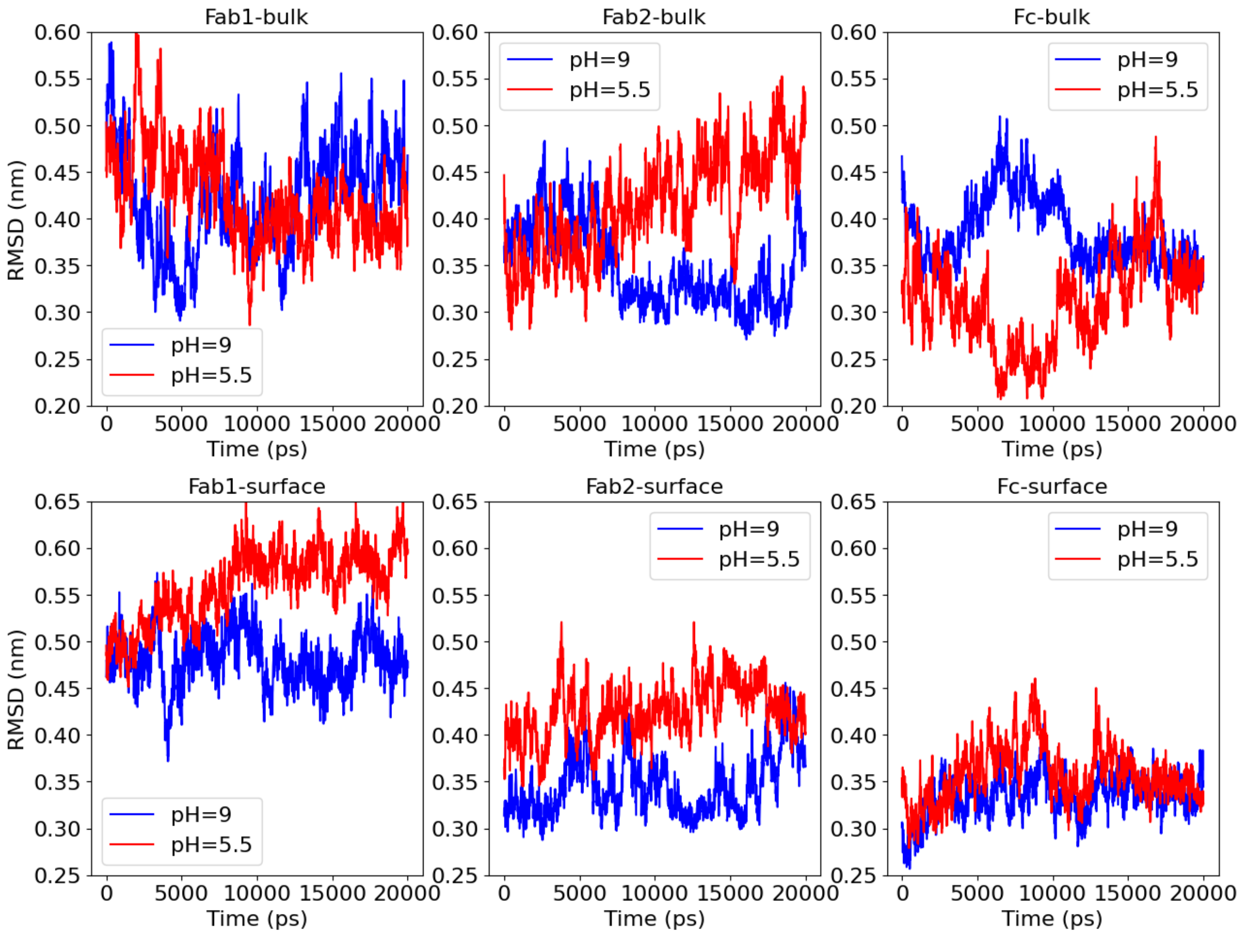

Figure S3. RMSD analysis of Fab1, Fab2 and Fc of COE-3 at pH 5.5 (Red) and pH 9 (Blue), both in bulk (Top) and in contact with the SiO_2 surface (Bottom).

SI 2

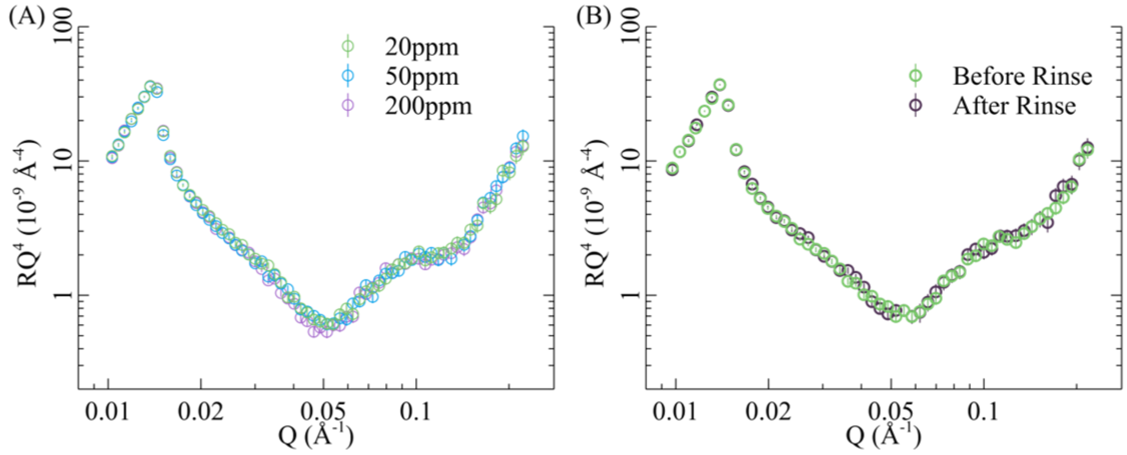

Figure S4. Neutron reflection profiles of COE-3 adsorption layer at different bulk concentrations and buffer rinse effects. (A) Neutron reflection profiles for COE-3 at bulk concentrations of 20, 50, and 200 ppm (0.02, 0.05, and 0.2 mg/ml), measured at surface saturation at 20°C. (B) Neutron reflection profile for COE-3 at a bulk concentration of 20 ppm (green circle) before and after a 20 ml buffer rinse at 2 ml/min at 20°C. The D_2O His buffer (ionic strength 25 mM, pD 5.8, equivalent to pH 5.5) was used for sample preparation and rinse.

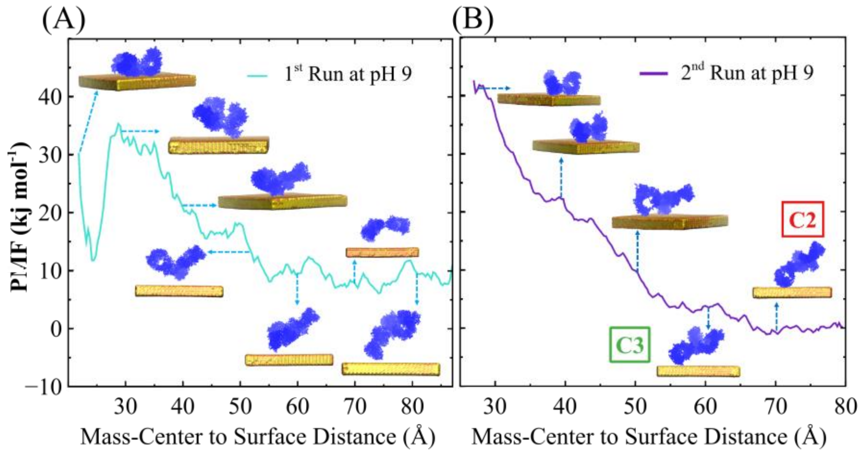

Figure S5. PMF profiles for the interaction between COE-3 and the SiO_2 surface in buffer (pH 9, ionic strength 25 mM). Results from two independent umbrella sampling runs are shown separately in panels (A) and (B). The corresponding COE-3 conformations at specific positions are labelled accordingly.

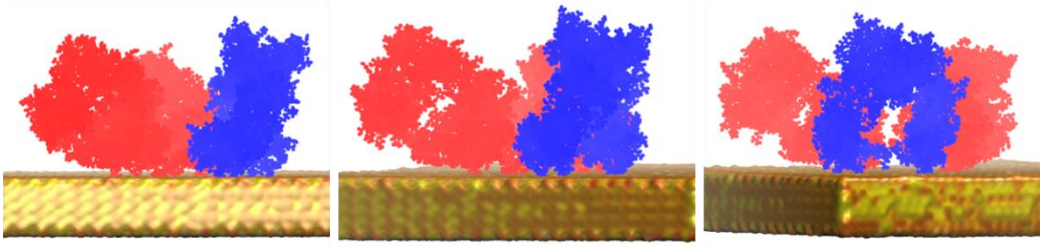

Figure S6. Views of Conformation C1 from different angles. The structure of Conformation C1 of COE-3 is shown from multiple viewpoints to provide a comprehensive understanding, with Fab and Fc fragments being distinguished by the colors red and blue, respectively.

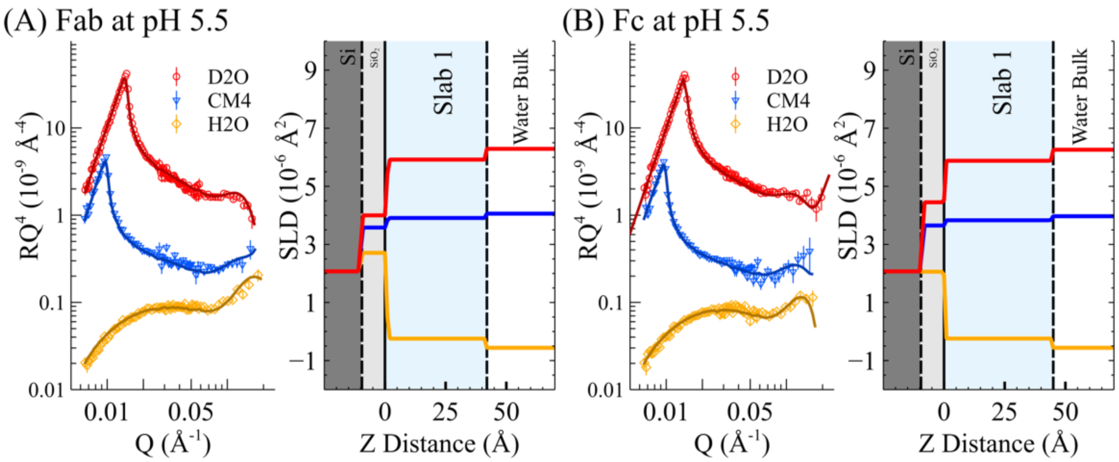

Figure S7. Simultaneous fitting of the three contrasts in parallel NR measurements of Fab (A) and Fc (B) adsorption layers at pH 5.5, IS = 25mM. The SLD profiles of the best fits are shown in the right panel of each graph.

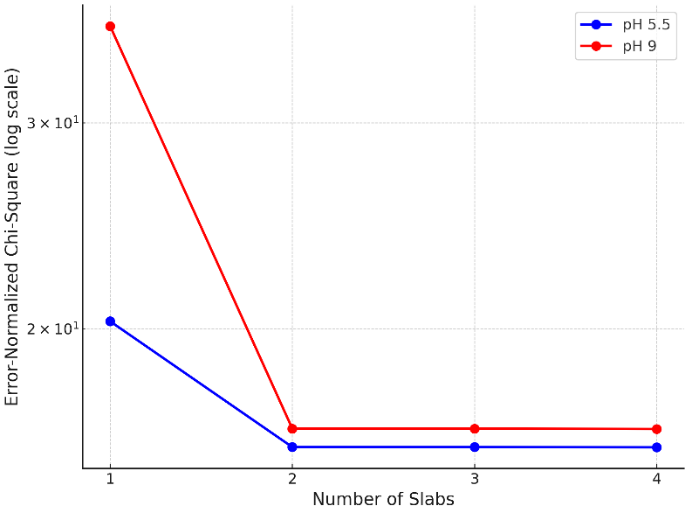

Figure S8. Error-Normalized Chi-Square as a function of the number of slabs used in the fitting model for the neutron reflection data analysis of pH 5.5 (blue) and pH 9 (red). The chi-square values show a significant reduction when increasing the number of slabs from 1 to 2, and further increases in the number of slabs provide only marginal improvements in the fit quality. It indicates that 2 is the optimal number of slabs used in the slab model for both pHs to achieve the best balance between model simplicity and goodness of fit.

REFERENCES

(1) Jo, S.; Kim, T.; Iyer, V. G.; Im, W. CHARMM-GUI: A Web-based Graphical User Interface for CHARMM. *J Comput Chem* **2008**, *29* (11), 1859–1865. https://doi.org/10.1002/jcc.20945.

(2) Olsson, M. H. M.; Søndergaard, C. R.; Rostkowski, M.; Jensen, J. H. PROPKA3: Consistent Treatment of Internal and Surface Residues in Empirical p *K* a Predictions. *J. Chem. Theory Comput.* **2011**, *7* (2), 525–537. https://doi.org/10.1021/ct100578z.

(3) Choi, Y. K.; Kern, N. R.; Kim, S.; Kanhaiya, K.; Afshar, Y.; Jeon, S. H.; Jo, S.; Brooks, B. R.; Lee, J.; Tadmor, E. B.; Heinz, H.; Im, W. CHARMM-GUI Nanomaterial Modeler for Modeling and Simulation of Nanomaterial Systems. *J. Chem. Theory Comput.* **2022**, *18* (1), 479–493. https://doi.org/10.1021/acs.jctc.1c00996.

(4) Lee, J.; Cheng, X.; Swails, J. M.; Yeom, M. S.; Eastman, P. K.; Lemkul, J. A.; Wei, S.; Buckner, J.; Jeong, J. C.; Qi, Y.; Jo, S.; Pande, V. S.; Case, D. A.; Brooks, C. L.; MacKerell, A. D.; Klauda, J. B.; Im, W. CHARMM-GUI Input Generator for NAMD, GROMACS, AMBER, OpenMM, and CHARMM/OpenMM Simulations Using the CHARMM36 Additive Force Field. *J. Chem. Theory Comput.* **2016**, *12* (1), 405–413. https://doi.org/10.1021/acs.jctc.5b00935.

(5) Kern, N. R.; Lee, J.; Choi, Y. K.; Im, W. *CHARMM-GUI Multicomponent Assembler for Modeling and Simulation of Complex Multicomponent Systems*; preprint; Biophysics, 2023. https://doi.org/10.1101/2023.08.30.555590.
